# Editorial: Salinity tolerance: From model or wild plants to adapted crops

**DOI:** 10.3389/fpls.2022.985057

**Published:** 2022-07-27

**Authors:** Quan-Sheng Qiu, Vanessa Jane Melino, Zhiguang Zhao, Zhi Qi, Crystal Sweetman, Ute Roessner

**Affiliations:** ^1^MOE Key Laboratory of Cell Activities and Stress Adaptations, School of Life Sciences, Lanzhou University, Lanzhou, China; ^2^College of Coastal Agricultural Sciences, Guangdong Ocean University, Zhanjiang, China; ^3^Academy of Plateau Science and Sustainability, School of Life Sciences, Qinghai Normal University, Xining, China; ^4^Center for Desert Agriculture and Division of Biological and Environmental Sciences and Engineering, King Abdullah University of Science and Technology (KAUST), Thuwal, Saudi Arabia; ^5^Key Laboratory of Herbage and Endemic Crop Biology, Ministry of Education, Inner Mongolia University, Hohhot, China; ^6^College of Science and Engineering, Flinders University, Bedford Park, SA, Australia; ^7^Research School of Biology, The Australian National University, Acton, ACT, Australia

**Keywords:** salt stress, signaling, response, GWAS, sequencing, crop, wild plants

Approximately 30% of irrigated land has salt-affected soil (Hopmans et al., 2021), equivalent to the area used to produce one-third of the world's food. Salinization and sodification are major soil degrading processes that reduce agricultural productivity, which along with the rapid depletion of groundwater reserves, is a major challenge to global food security (Hopmans et al., 2021). Given that genetic variation is the basis for crop improvement, there are many avenues for researchers to exploit, from identifying traits related to salt tolerance to genetic control of traits using locally adapted plants (crop wild relatives and landraces), genetic populations or mutant variants (Morton et al., 2019; Bohra et al., 2022). There is also great potential to compare and translate findings of genetic regulation of salt stress responses from model plant species to crops through genome editing and gene modifying techniques.

Salt tolerance is a genetically complex and can be dissected into the contributing traits and mechanisms (Munns and Tester, 2008; Roy et al., 2014). This can include traits such as yield and fruit or grain quality and the breakdown into component traits, such as transpiration, photosynthesis, nutrient uptake, senescence, ROS scavenging, ion compartmentation and ion transport, amongst others (Morton et al., 2019). Plants can respond to salt stress in various ways, the differences can be analyzed in comparative physiological and genetic studies. Salinity can also reduce the osmotic and water potential of the growth medium, inhibiting water uptake (Roy et al., 2014). Here, we are interested in examining novel findings that will assist us in understanding genetic determinants of salt stress responses and adaptation using a variety of physiological, genomic and genetic techniques.

This Research Topic aims to present novel research findings on salinity tolerance in plants, especially crops. The scope of this topic includes: (i) understanding physiological and molecular mechanisms behind plant sensing, signaling and responses to salt stress; (ii) conferring salinity tolerance to plants, especially crops through the introduction of genes and regulators of responses and/or adaptation to salt stress; (iii) identifying salinity tolerance traits in wild progenitors and landraces and/or translating this into crops.

## Plant signaling and responses to salt stress

Xie et al. reviewed the structure and function of the Na^+^/H^+^ antiporter and the physiological process of Na^+^ transport controlled by the SOS signaling pathway. They carried out phylogenetic analysis of SOS1 proteins in plants, and implied the specificity of salt tolerance mechanisms from model plants to crops under salt stress. They summarized the complexity of the regulatory network for adaptation to salt tolerance, and the feasibility of coping strategies of plants that could be used for genetic improvement under salt stress.

Mao et al. found that overexpression of *NtCBL5A* encoding the calcineurin B-like protein caused salt hypersensitivity with necrotic lesions on leaves. Leaves of the *NtCBL5A*-OE lines tended to curl and accumulated high levels of ROS under salt stress. Transcriptome profiling showed that many immune response-related genes are upregulated and photosynthetic machinery-related genes are downregulated in the leaves of the *NtCBL5A*-OE lines under salt stress. Overexpression of *NtCBL5A* interferes with salt stress responses of tobacco plants and leads to Na^+^-dependent leaf necrosis by enhancing the sensitivity of transgenic leaves to Na^+^.

Song et al. report that the phytohormone jasmonic acid (JA) impairs plant salt tolerance by repressing *CAT2* expression in an MYC2-dependent manner. They found that exogenous JA application decreased salt tolerance while the *jar1* mutant showed enhanced salt tolerance. JA enhanced salt-induced H_2_O_2_ accumulation, while treatment with H_2_O_2_-scavenger glutathione compromised such effects of JA. JA repressed *CAT2* expression in salt-stressed wild-type plant but not in *myc2*, a mutant of the master transcriptional factor MYC2 in JA signaling.

Lin, Zhou et al. examined methylom responses to salt stress from *Arabidopsis* natural accessions. It has been suggested that the epigenetic mechanism such as DNA methylation can mediate plant responses to salt stress but analysis of genome-wide methylation dynamics under salt stress remains limited for multiple genotypes. This study indicates that, across different genetic backgrounds, methylation changes may have convergent functions in post-transcriptional, physiological, and phenotypic modulation under salt stress. These convergent methylation dynamics across accession may be autonomous from genetic variation or due to convergent genetic changes.

Zhang, Zhou et al. performed genome-wide association studies (GWAS) of salt tolerance in *Brassica napus*. 16 salt tolerance coefficients (STCs) were applied to investigate the genetic basis of salt tolerance of *B. napus*. 31 salts stress-related QTLs were mapped, and 177 and 228 candidate genes related to salt tolerance were detected at germination and seedling stages, respectively. Overexpression of two candidate genes, *BnCKX5* and *BnERF3*, increased the sensitivity to salt and mannitol stresses at the germination stage. This study demonstrated that GWAS is a feasible method to dissect the genetic basis of salt tolerance in *B. napus*, which provides valuable loci for improving salt tolerance of *B. napus*.

Zhang, Dai et al. examined whether H_2_O_2_ pretreatment improves salt tolerance. They demonstrated that pretreatment with H_2_O_2_ enhanced salt tolerance of Arabidopsis seedlings, as revealed by lower Na^+^ levels, higher K^+^ levels, and improved leaf K^+^ /Na^+^ ratio. H_2_O_2_ pretreatment improved the membrane properties by reducing the relative membrane permeability and malonaldehyde content as well as enhancing the activity of antioxidant enzymes. The transcription data show that H_2_O_2_ pretreatment induced the expression of cell cycle, redox regulation, and cell wall organization-related genes in Arabidopsis, which may accelerate cell proliferation, enhance tolerance to osmotic stress, maintain the redox balance, and remodel the cell walls of plants in subsequent high-salt environments.

Wu et al. examined the role of *Vitis vinifera* VviNPF2.2 in modulating shoot anion concentration in transgenic Arabidopsis. Grapevines are sensitive to salt-forming ions, particularly chloride (Cl^−^) when grown in saline environments. Molecular components underlying Cl^−^-exclusion in *Vitis* species remain largely unknown. In this paper, two nitrate/peptide transporter family (NPF) members *VviNPF2.1* and *VviNPF2.2* were isolated, which are homologous proteins localized in the plasma membrane of Arabidopsis protoplasts. Both were expressed primarily in grapevine roots and leaves and were more abundant in a Cl^−^-excluding rootstock compared to a Cl^−^-accumulator. Quantitative PCR revealed that *VviNPF2*.*1* and *VviNPF2.2* expression was downregulated by high ${\text{NO}}_{3}^{-}$ resupply post-starvation. Constitutive expression of *VviNPF2.2* exclusively in the root epidermis and cortex reduced shoot [Cl^−^] after a 75 mM NaCl treatment. Higher expression levels of *VviNPF2.2* correlated with reduced Arabidopsis xylem sap [${\text{NO}}_{3}^{-}$] when not salt stressed. VviNPF2.2, through its role in the root epidermis and cortex, could, therefore, be beneficial to plants under salt stress by reducing net shoot Cl^−^ accumulation.

Ji et al. examined the role of plant growth regulators in modulating root architecture and tolerance to high-nitrate stress in tomato. The roles of four common exogenously applied plant growth regulators (MT, melatonin; SA, salicylic acid; HA, humic acid; SNP, sodium nitroprusside) in regulating tomato growth were studied under high-nitrate stress. They show that all four growth regulators improve tomato tolerance under high nitrate, with MT and SNP produced the strongest effects. MT enhanced root growth while SNP enhanced above-ground growth. An enhancement of root vitality and metabolism, improved integrity of root cell membranes, and an increase in antioxidant enzyme activities were found, but regulatory mechanisms were different for each growth regulator. Results show that the application of MT and SNP can improve growth of tomato in intensive vegetable production under high-nitrate stress.

## Salt tolerance in crops

Lin, Wang et al. examined the role of a cell wall-associated kinase OsWAK112 in regulating salt stress response in rice. Overexpression of *OsWAK112* in rice and *Arabidopsis* decreased survival rate under salt stress, while knocking down the *OsWAK112* in rice increased survival. OsWAK112 possesses kinase activity and plays a negative role in response to salt stress. OsWAK112 interacts with S-adenosyl-L- methionine synthetase (SAMS) 1/2/3, and promotes OsSAMS1 degradation under salt stress. SAMS and ethylene are decreased in *OsWAK112*-overexpressing plants under salt stress. These results indicate that OsWAK112 negatively regulates plant salt responses by inhibiting ethylene production, possibly *via* binding with OsSAMS1/2/3.

Lu et al. studied the effects of exogenous melatonin on alleviating alkaline stress in rice. Saline-alkali stress seriously restricts rice growth and production in northern China. Melatonin (N-acetyl- 5-methoxytryptamine, MT) mediates a variety of physiological processes in rice and protects rice from abiotic stress. The mechanism of melatonin-mediated alkaline tolerance is largely unknown. The results showed that the expression levels of MT synthesis genes were induced under treatments of exogenous MT and alkaline stress. MT plays a role in scavenging reactive oxygen species, reducing lipoxygenase activity and malondialdehyde contents. MT pretreatment promoted the accumulation of free proline, sucrose, and fructose by regulating *OsP5CS, OsSUS7*, and *OsSPS1* expression and increasing chlorophyll contents via upregulating the expression of chlorophyll synthesis-related genes. Ultimately, the alleviating effect of exogenous melatonin on alkaline stress was reflected in increasing the leaf relative water content and root-shoot ratio as well as reducing the wilt index of leaf tips.

Chen, Huang et al. examined the influence of salt stress on the metabolomic profiling of Dongxiang wild rice (DXWR), which is a rice germplasm resource for understanding and improving salt tolerance in rice. They found that amino acids and nuclear glycosides were upregulated, while carbohydrates and organic acids were downregulated under salt stress. They further found that the change in L-Asparagine was the highest between salt-tolerant and non-salt-tolerant progenies under salinity, indicating that L-Asparagine could be used as an index in evaluating salt tolerance of rice varieties. These results demonstrate the significant role of amino acids in salt tolerance in rice.

Chen, Hu et al. performed genome-wide association study (GWAS) for salt-induced phenotypic and physiologic responses in rice at seedling and reproductive stages. They evaluated salt tolerance (ST) levels of 220 rice accessions at both seedling and reproductive stages. A total of 214 SNPs related to 251 genes, which are associated with 16 ST-related indices, were detected at both stages. 82 SNPs with low frequency favorable (LFF) alleles in the population were proposed to hold high breeding potential in improving rice ST. 54 rice accessions collectively containing all these LFF alleles were identified as donors of these alleles. 38 candidate genes were suggested to be involved in the regulation of rice ST. This study provides valuable information for further characterizing ST-related genes and for breeding ST varieties across whole developmental stages through marker-assisted selection (MAS).

Kong et al. compared the transcriptome of the rice varieties *indica* and *japonica* to understand their differences in salt tolerance. Two rice genotypes, RPY geng (*japonica*, tolerant variety) and Chao 2R (*indica*, susceptible variety), were used in this study. 7208 and 3874 differentially expressed genes (DEGs) were identified under salt stress in Chao 2R and RPY geng, respectively. The expression of normal life process genes in Chao 2R were affected under salt stress, but RPY geng regulated the expression of multiple stress-related genes to adapt to salt stress. They highlighted important pathways and transcription factors (TFs) related to salt tolerance in RPY geng specific DEGs sets based on MapMan annotation and TF identification. Through Meta-QTLs mapping and homologous analysis, they identified 18 salt stress-related candidate genes (RPY geng specific DEGs) in 15 Meta-QTLs. These findings have assisted with identification of target genes to facilitate gene editing in order to enhance salt tolerance in rice.

Thummala et al. performed the whole-genome sequencing of an elite rice restorer line KMR3 (salinity-sensitive) and its salinity-tolerant introgression line IL50-13, a popular variety of coastal West Bengal, India, to identify candidate genes for high yield and salinity tolerance in rice. Functional enrichment analysis of the protein-coding genes with unique InDels identified GO terms involved in protein modification, ubiquitination, deubiquitination, peroxidase activity, and antioxidant activity in IL50-13. Linoleic acid and alpha-linolenic acid metabolism, and circadian rhythm pathways were enriched in IL50-13. Polymorphism was observed in the coding, intron, and untranslated regions of the genes on chromosomes 1, 2, 4, 7, 11, and 12. IL50-13 is an introgression line that was derived from KMR3 x *Oryza rufipogon*. In this study, KMR3 was used as the control line and IL50-13 as the experimental line. Thus, genes showing polymorphism between the two genomes were considered as sequence-based new candidates derived from *O. rufipogon* for conferring high yield and salinity tolerance in IL50-13 for further functional studies.

Guo et al. identified two novel loci, GmSALT3 and GmSALT18 genes, associated with soybean sensitivity to salt stress. NY36-87 is a wild soybean germplasm with high salt tolerance. In this study, two F_2:3_ mapping populations derived from NY36-87 and two salt-sensitive soybean cultivars, Zhonghuang39 and Peking, were used to map salt tolerance-related genes. They mapped a salt tolerance locus on chromosome 03 in F_2:3_ population Zhonghuang39 × NY36-87, in which the known gene *GmSALT3* co-segregated with the salt tolerance locus. In the F_2:3_ population of Peking × NY36-87, the dominant salt tolerance-associated gene was detected and mapped on chromosome 18, where they named the gene *GmSALT18*. These findings reveal that the tolerant wild soybean line NY36-87 contains salt tolerance-related genes *GmSALT3* and *GmSALT18*, providing genetic material and a novel locus for breeding salt-tolerant soybean.

Sultana et al. found that seed priming and foliar spray of 10 mg L^−1^ coumarin, a potent phenolic compound, could significantly alleviate salinity-induced effects in *Sorghum bicolor*, including plant growth, biochemical attributes and photosynthetic efficiency.

## Salinity tolerance in wild plants

Li, Duan et al. reviewed the adaptative mechanisms of halophytic *Eutrema salsugineum* ecotypes when exposed to saline environment. Salt cress (*E. salsugineum*), an Arabidopsis-related halophyte, is naturally adapted to various harsh climates and soil conditions, thus it is considered as a desirable model plant for deciphering mechanisms of salt and other abiotic stresses. This review summarizes studies on the morphology, physiology, genomics, gene regulation, protein and metabolite profiles of salt cress under salt stress. They emphasize the latest advances on the adaptive evolution when encountering saline environments, and epigenetic regulation, and discuss the mechanisms underlying salt tolerance in salt cress. They discussed the existing questions and opportunities for future research with Eutrema.

Li, Zhao et al. examined the role of the *EsMYB90* gene from halophytic *Eutrema salsugineum* in improving growth and antioxidant capacity of transgenic wheat under salt stress. They found that wheat JW1 (a spring bread wheat) overexpressing *EsMYB90* showed longer roots and higher fresh weight. The transgenic wheat plants displayed higher peroxidase and glutathione S-transferase activity as well as lower malondialdehyde content and accumulated less reactive oxygen species (ROS) under salt stress. Transcriptome analysis revealed that the *EsMYB90* gene affected the expression of considerable amounts of stress-related genes that were involved in phenylpropanoid biosynthesis and antioxidant activity in transgenic plants subjected to NaCl treatment. The upregulated expression genes under salt stress were associated with the antioxidant enzymes POD- and GST-encoding genes. *EsMYB90* is suggested to activate the transcription of *TaANS2* and *TaDFR1* genes that are encoding enzymes of anthocyanin biosynthesis. The results indicate that the *EsMYB90* gene plays a crucial role in preventing wheat seedlings from oxidative stress *via* enhancing the accumulation of non-enzymatic flavonoids and activities of antioxidative enzymes.

Ma et al. performed global analyses of mRNA alternative polyadenylation (APA) in Arabidopsis, a salt-sensitive species, and its halophytic, salt-tolerant relative *Eutrema salsugineum* in response to salt stress. Percentage of APA genes and distal poly (A) sites significantly increased in Arabidopsis but not in Eutrema. In addition, the transcripts with upregulated poly (A) sites showed different enriched pathways in both species: Arabidopsis prefers plant hormone signal transduction, starch and sucrose metabolism, and fatty acid elongation, while Eutrema prefers biosynthetic and metabolic pathways. Overall, the study demonstrated that the ability to maintain RNA maturation stability is an important means for plants to be adapted to salt stress.

Zhu et al. examined the role of *NtSOS2* from the halophyte *Nitraria tangutorum* in enhancing salt tolerance in *Arabidopsis*. The Salt Overly Sensitive (SOS) signaling pathway is key in responding to salt stress in plants. The role of *SOS2* has not been reported in *N. tangutorum*. In this paper, the *NtSOS2* gene was cloned, and phylogenetic analyses showed that *NtSOS2* is homologous to the SOS2 of *Arabidopsis* and rice. *NtSOS2* wa*s* expressed in the cytoplasm and cell membranes and was induced by salt stress. Expression of *NtSOS2* in *Arabidopsis* reduced leaf mortality and improved germination rate, biomass and root growth under salt stress. Also, expression of *NtSOS2* affected the expression of ion transporter-related genes and could rescue the phenotype of *sos2-1* under salt stress. All these results indicate that *NtSOS2* plays an important role in salt tolerance.

Ahmad et al. conducted transcriptomic and metabolomic analysis of *Solenostemma argel Hayne* roots and leaves, a desert plant, in response to salt stress. There are 730 and 927 differentially expressed genes identified in the roots and leaves. There are 45 and 56 metabolites showing significant abundance changes in the roots and leaves, respectively, which are associated with antioxidant ability and osmotic adjustment.

Marriboina et al. systemically characterized physiological and protein responses of a potential biofuel tree *Pongamia pinnata* to salt stress. The study suggested that the tree could maintain its leaf morphology through limiting net photosynthetic rates and gas exchange. Under salt stress, the tree might recruit high-abundance proteins related to flavonoid biosynthesis, seed storage and carbohydrate metabolism to protect its roots from the salt toxicity.

Li, Lin et al. examined the anatomical characteristics and conducted proteomic analysis of three different ecotypes of reeds in the Badanjilin desert of Northwest China, including the typical swamp reed as a control and the two terrestrial, dune reed (DR) and heavy salt meadow reed (HSMR), adapted to the water deficit and saline environment. They found that both DR and HSMR have evolved C4-like photosynthetic and anatomical characteristics. In addition, it was suggested that DR and HSMR could regulate proteins associated with photosynthesis, lipid metabolism and gene transcription to adapt to the harsh desert environment.

Chen, Jin et al. demonstrated that two wild plant species used different strategies to cope with saline-alkali soil conditions in the Hulun Buir Grassland. *Suaeda salsa* primarily accumulated K^+^ and Ca^2+^ and was able to limit Na^+^ uptake and facilitate Na^+^ efflux. Mn^2+^ was enriched in the *Puccinellia tenuiflora* roots and could activate the Mn^2+^-SOD activity, which increases the plant's resistance to the stress. In addition, they found that under the saline-alkali condition, *S. salsa* had high levels of the stress signaling molecule C6C1 and C6C3C6, whilst *P. tenuiflora* accumulated C6C3 to eliminate the free radicals.

Ounoki et al. found that low level salinity stress promoted stem-derived adventitious root formation of freshly cut shoots of spearmint without any visible limitation on its growth. Moreover, NaCl at 50 mM and above strongly inhibited adventitious root formation, photosynthesis efficacy and disrupted the chlorophyll-protein complexes as well as chloroplast structure. However, the salt stress has no significant effects on the essential oil composition.

Hashemi-Petroudi et al. studied the genome of *Aeluropus littoralis*, a halophile grass. The use of wild plant species or their halophytic relatives has been considered in plant breeding programs to improve salt and drought tolerance in crop plants. *A. littoralis* serves as a halophyte model for identification and isolation of novel stress adaptation genes. In this paper, the authors described the genome sequence and structure of *A. littoralis* analyzed by whole genome sequencing and histological analysis. The chromosome number was determined to be 20 (2n = 2X = 20), the absence of a B chromosome, and the genome size calculated to be 354 Megabasepairs. This genomic information will support the functional investigation and application of novel genes improving salt stress resistance in crop plants.

## Author contributions

All authors contributed to the Research Topic and wrote and edited the Editorial.

## Conflict of interest

The authors declare that the research was conducted in the absence of any commercial or financial relationships that could be construed as a potential conflict of interest.

## Publisher's note

All claims expressed in this article are solely those of the authors and do not necessarily represent those of their affiliated organizations, or those of the publisher, the editors and the reviewers. Any product that may be evaluated in this article, or claim that may be made by its manufacturer, is not guaranteed or endorsed by the publisher.
